# Lesson-drawing in tobacco control: A qualitative study of stakeholder perceptions in five North-Western European countries

**DOI:** 10.18332/tpc/161999

**Published:** 2023-04-26

**Authors:** Thomas G. Kuijpers, Anton E. Kunst, Marc C. Willemsen

**Affiliations:** Department of Health Promotion, CAPHRI School for Public Health and Primary Care, Maastricht University, Maastricht, The Netherlands; Trimbos Institute, Netherlands Institute of Mental Health and Addiction, Utrecht, The Netherlands; Department of Public Health, Academic Medical Center, University of Amsterdam, Amsterdam, The Netherlands

**Keywords:** advocacy, lesson-drawing, policy learning, family of nations, tobacco control

## Abstract

**INTRODUCTION:**

The theory of ‘family of nations’ posits that countries draw policy lessons predominantly from similar countries. Lesson-drawing in tobacco control has, however, been primarily studied in the ‘English-speaking’ family. We examined in five diverse North-Western European countries whether the government engages in lesson-drawing regarding best practices in tobacco control, which countries they look at for guidance, and why these were chosen as a reference.

**METHODS:**

Perceptions of 29 policy participants from civil society and government were assessed by means of interviews conducted in Belgium, Finland, Germany, Ireland, and the Netherlands. Relevant excerpts were grouped according to country and a bottom-up thematic analysis was performed.

**RESULTS:**

The tobacco control instruments described by the policy participants were tobacco marketing bans (display ban and plain packaging) and smoke-free policies. German interviewees stated that the German federal government is not inclined to engage in foreign lesson-drawing. All other governments were perceived to look at Australia for lessons because of its global leadership in tobacco control. At the same time however, lessons from Australia were easily dismissed because it is an ‘island’ and far away. Irish interviewees observed their government to primarily look at other English-speaking countries. Governments in Belgium, Finland and the Netherlands were observed to primarily look at nearby European countries for lessons.

**CONCLUSIONS:**

Countries in North-Western Europe seem to draw policy lessons based on proximity and similarity to other countries concerning marketing bans and smoke-free policies. Proponents of tobacco control may use these findings to facilitate effective lesson-drawing in their countries.

## INTRODUCTION

Countries have responded differently to the public health problems caused by tobacco consumption^1-3^. Such international differences in policy responses offer ample opportunity for countries to draw lessons in tobacco control from other countries^4,5^. Lesson-drawing, or policy learning, is a voluntary type of policy transfer in which lessons are drawn from a country’s past or from other countries^4-6^. The main objective of lesson-drawing from other countries is to use cross-national policy experience as a source of policy advice^7^.

The most critical question, when countries draw lessons from other countries, is whether a certain policy instrument (or policy) is transferable from the ‘exporting’ country to the ‘importing’ country^8^. During this ‘prospective evaluation’, the national context in which a policy instrument is implemented needs to be taken into account. Proponents of a certain policy instrument may argue that it is transferable, pointing to similarities between national contexts – thus hoping to increase political support for the instrument^9^. Opponents may argue that an instrument cannot be transferred because national contexts are too different^9^. Given such debates, knowing which considerations play a role in accepting a policy lesson from another country can enable tobacco control advocates to focus on specific countries and contextual factors when promoting lessons from elsewhere^10^.

The perceived transferability of policy instruments is likely higher when importer and exporter countries are more similar to each other. The ‘family of nations’ theory posits that countries can be clustered on the basis of similarities in their public policy profiles^11,12^. Studlar^13^ analyzed patterns of tobacco control policy adoption across fourteen countries over time, and concluded that three overlapping yet distinctive groups with similar policy profiles could be distinguished: an ‘Anglo-American’, a ‘Scandinavian’, and a ‘European Union’ group. He observed policy convergence within these families and pointed to lesson-drawing as a key explanation. Other scholars who study diffusion of tobacco control policies have also used the concept of lesson-drawing as an explanation for observed patterns of policy adoption across various jurisdictions^14-16^. Other tobacco control scholars adopt a more qualitative approach and study single cases of policy transfer between two countries or jurisdictions. In these instances, lesson-drawing is often used in addition to other theories of policymaking, highlighting the notion that there are multiple influences relevant to the process that eventually leads to policy adoption^17-21^.

With two exceptions^13,16^, scholars in tobacco control have only focused on lesson-drawing between English-speaking countries, which have the most comprehensive tobacco control policies enacted, both in Europe and worldwide^1^. Lesson-drawing has hardly been studied across more culturally diverse groups of countries (Table 1). In this study, we will assess perceptions of policy participants across five different North-Western European countries to examine: 1) whether they perceive their national government to draw lessons from other countries, 2) from which countries their governments are observed to learn lessons, and 3) why they think that lessons are mostly drawn from these countries.

**Table 1:** Characteristics of the included countries in 2017*

*Country*	Population size** (millions)	*Smoking prevalence (%)*	*Language*	*Most influence on tobacco control policymaking*	*Tobacco control frame in politics*	*Health advocacy institutions*	*Tobacco industry economic presence*	*Public health policy frameworks*	*Interpretation FCTC Art. 5.3*
**Finland**	5.5	20	Finnish	Health organizations	Health	Developed	Largely gone	Endgame strategy	Strict
**Ireland**	4.8	19	English	Health organizations	Health	Developed	Largely gone	Endgame strategy	Strict
**The Netherlands**	17.1	1	Dutch	Unclear	Individual choice/paternalistic government	Developed	Largely gone	No	Strict
**Belgium**	11.4	19	Dutch/French	Unclear	Individual choice/paternalistic government	Developed	Largely gone	No	In terms of transparency
**Germany**	82.8	25	German	Tobacco industry and business	Private problem/no discussion	Weak	Manufacture and production	No	In terms of transparency

* Adapted from Kuijpers, Kunst and Willemsen^22^.

** European Union: First population estimates EU population up to almost 512 million at 1 January 2017, in Eurostat News Release 2017.

## METHODS

### Project background

This study was part of a larger European study conducted in seven EU countries: Belgium, Finland, Germany, Ireland, Italy, the Netherlands, and Portugal. The SILNE-R project assessed how smoking prevention strategies were adopted and implemented within seven countries, at national, municipal, and school levels, and how the process of adoption and implementation varied between countries, cities, and schools.

### Stakeholder selection

National representatives of the SILNE-R project provided a list of key stakeholders relevant to national tobacco control policymaking, in some cases with help of national key informants known to the project. Initially, 40 policy participants were invited, of whom 26 agreed to be interviewed (response rate of 65%). Almost all the non-responses were observed from Members of Parliament and civil servants. Twenty-six interviews with twenty-nine policy participants [three interviews with two policy participants in Germany (2) and Ireland (1)] were ultimately conducted. Policy participants worked inside and outside government and were purposefully selected in Belgium, Finland, Germany, Ireland, and the Netherlands. Portugal was excluded from the study due to the continued non-response of stakeholders. Interviews from Italy, included in the original study^22^, provided too little information on lesson-drawing and were excluded from the analysis. This was because the focus of the original study was to get information on the policy process surrounding a display ban of tobacco products and the part on lesson-drawing/policy-learning was one of ten interview topics. Each type of stakeholder was successfully interviewed in every country, except for a Dutch civil servant (because of the salience of the policy issue at that time).

At least five interviewees were chosen as a minimum per country, providing different perspectives of the national policy environment: a civil servant, a member of parliament, an academic expert, an employee of a national cancer fund or other health NGO, and an employee of a national tobacco control alliance, when such an alliance existed (see Table 2 for a complete list of all stakeholders per country). The interviewees were active participants in tobacco control policy development in their countries.

**Table 2:** List of policy participants per country

Country	*Policy participants*
**Belgium**	Civil servant
	Member of Parliament (Opposition)
	Cancer fund employee
	Academic expert 1
	Academic expert 2
	Civil society organization employee
**Finland**	Civil servant
	Member of Parliament (Opposition)
	Cancer fund employee
	Academic expert
	Tobacco Control Alliance network employee
	Enforcement agency employee
**Germany**	Civil servant
	Member of Parliament (Coalition)
	Assistant to Member of Parliament
	Cancer fund employee
	Academic expert
	Civil society organization employee 1
	Civil society organization employee 2
**Ireland**	Civil servant
	Member of Parliament (Senate)
	Cancer fund employee
	Academic expert
	Alliance network employee
**The Netherlands**	Member of Parliament (Opposition)
	Cancer fund employee
	Academic expert
	Tobacco Control Alliance network employee 1
	Tobacco Control Alliance network employee 2

### Interview topics

The interviews were semi-structured and consisted of ten different interview subjects to be discussed (see Supplementary file). One part focused on lesson-drawing from other countries with regard to a tobacco display ban, which was the focus of the original study^22^. However, during this part of the interview, stakeholders frequently referred to other tobacco control policy instruments (e.g. plain cigarette packaging and smoke-free policies) to illustrate which countries were looked at for lessons in tobacco control, and why.

The first question in this part of the interview was: ‘Did the government look abroad to other country experiences with a point-of-sale display ban?’. Follow-up questions were: ‘What countries?’ and ‘Why these countries?’. Interviews were conducted in Dutch (Belgium and the Netherlands), English (Finland and Ireland), and German (Germany). They lasted on average 64 minutes and were transcribed verbatim. Relevant excerpts were translated from Dutch or German to include in the manuscript. Interviews were conducted between January and August 2017.

### Analysis

TGK performed the initial data-analyses and discussed preliminary findings and themes during weekly sessions with MCW and during monthly sessions with both MCW and AEK.

All relevant excerpts from the transcripts were grouped according to the five countries under study. Per country, one or more ‘example countries’ were mentioned by respondents. TGK then identified themes within the countries (i.e. arguments or reasons to draw or dismiss lessons) and noted whether, according to the interviewee, these lessons were likely to be accepted or not. Subsequently, these themes (arguments/reasons to draw or dismiss lessons) were compared across the five countries to discover patterns and similarities between these countries. Preliminary themes were discussed with the other authors and further refined in an iterative manner.

## RESULTS

### Belgium

Interviewees in Belgium indicated that, in relation to plain packaging, their government looked at Australia, France, and the United Kingdom. The main cited reason to draw lessons from these countries was that policymakers wanted to see how the implementation of that instrument worked out in countries where such a measure had already been implemented:

*‘One of the conditions that the minister [of health] attaches to the adoption of neutral packages […] is how the implementation of neutral packages goes in France and the United Kingdom.’* (Belgium, interviewees)

Belgium, civil society advocate perceived reasons to dismiss lessons were: the country is an ‘island’ (Australia and the United Kingdom), and smoking prevalence remains relatively high despite a restrictive tobacco control regime (France):

*‘Yes, she [minister of health] uses all kinds of excuses: […] “Yes, maybe it was found to be efficient there, but Australia is an island and we need data from a country that is not an island”. So, they are waiting for results from France where the measure has just been implemented and that may take a while.’* (Belgium, civil society advocate)

‘France is a bizarre country. Because it is a country that takes many measures against smoking, but the measures oddly enough have much less effect than in other countries.’ (Belgium, academic expert 1)

### Finland

According to the interviewees, the Finnish government looks at Australia (plain packaging) and other Nordic countries (display ban and plain packaging). Perceived reasons to look at Australia were that it is a global leader in tobacco control and they have the same endgame goal (i.e. a smoke-free society):

*‘Currently our tobacco legislation is very advanced, but for many details some countries have gone further than Finland, so we must look at their good examples. Nowadays there is clear evidence from Australia that it [plain packaging] is a feasible and useful thing.’* (Finland, Member of Parliament)

Perceived reasons to look at other Nordic countries were that the countries are close, there is a shared historical collaboration with established communication channels (through the Nordic council), a shared culture, and similar political systems. A cited reason to dismiss lessons from Australia, was that it is far away. There were no perceived reasons to dismiss lessons from other Nordic countries:

*‘We are living in a similar area [as other Nordic countries] and we have a similar culture. We have different nationalities but it’s closer than Australia. Of course, concerning plain packaging, we take the evidence from Australia. But if it’s closer, it’s easier to convince.’* (Finland, civil servant)

### Germany

German respondents do not perceive their government to engage in lesson-drawing from other countries. In contrast to the German NGO community, the German government is not perceived to look abroad for policy lessons in tobacco control. The main perceived reason is that there is no political majority for governmental intervention in tobacco control:

*‘It is always interesting what Australia does, France also does some things with which we are engaged more closely, the United Kingdom is also much more progressive. These are the wonderfully interesting actors. However, the decisive factor is not knowing about what you could possibly do, but the main question is whether there is a political majority [for tobacco control] in a country, and in Germany there isn’t.’* (Germany, civil servant)

Observed reasons to dismiss lessons from Ireland, as proposed by the NGO community regarding smoke-free legislation, were that it is distant, it has a smaller country size, and a different language:

*‘Our approach is always to present the evidence, and there is so much evidence from other countries. That is the advantage of always being the last, or one of the last ones, to introduce something: we can always show how well the legislation works in other countries. […] But it just doesn’t work. The problem is just that the political will is not there.’* (Germany, civil society advocate 1)

*‘Germany is a big country and when you compare Germany with Ireland, it is not a comparison. You must compare it with France. That would be respected, but not with various small countries.’* (Germany, civil society advocate 2)

### Ireland

The Irish government was observed to look at Australia (plain packaging), Canada (display ban), and the United Kingdom (plain packaging). Perceived reasons to draw lessons from these countries were that they were global leaders in tobacco control (all countries) and that there are historical connections to Australia and Canada. There were no perceived reasons to dismiss lessons from these countries:

*‘Yes, at the moment Australia is leading the way globally in terms of tobacco control. I think Canada is also very strong. But having said that, the United Kingdom and Ireland from a European perspective are at the top of the [Tobacco Control] Scale in terms of tobacco control measures. So, we do try and lead the way and try to push forward leading initiatives.’* (Ireland, civil society advocate)

*‘Why Australia? Because they were the first to bring in plain packaging.’* (Ireland, Member of Parliament)

### The Netherlands

Interviewees stated that the Dutch government looks at Australia (plain packaging and smoke-free) and England (display ban, plain packaging, and smoke-free). Australia was looked at because of its global leadership in tobacco control. However, at the same time, lessons from Australia were dismissed because it is perceived as being ‘far away’ and an isolated ‘island’, subjected to ‘different natural laws’:

*‘We would rather not [take Australia as an example], because it is far away and an isolated island with different natural laws compared to Europe.’* (The Netherlands, civil society advocate 1)

*‘In general, European countries are preferred over countries somewhere else in the world, because countries outside Europe are less comparable. Western European countries are most preferred.’* (The Netherlands, civil society advocate 1)

Perceived reasons to draw lessons from England were that it has a comparable political system, similar tobacco control progressiveness, it is considered a reliable country, and there are good scientific policy evaluations available. There were no perceived reasons not to look at England:

*‘[The Netherlands and England] generally have comparable legislative systems ... or at least fairly similar. Enforcement works pretty much the same, it has to be organized well and an exception must be properly codified.’* (The Netherlands, civil society advocate 2)

## DISCUSSION

All governments except the German government were perceived to engage in lesson-drawing from other countries. The other governments were all perceived to look at Australia for lessons because of its global leadership in tobacco control. Australia had implemented policy instruments that were being discussed in the policy environment of several European countries, particularly plain packaging. However, except for the Irish government, governments in all countries were found to have similar reasons to ultimately dismiss policy lessons from Australia: because it is ‘far away’ and an ‘island’.

Governments in Belgium, Finland and the Netherlands seemed, in contrast to Ireland, more inclined to look at European countries nearby for lessons in tobacco control. When providing reasons why countries are chosen as an example, interviewees in Finland and the Netherlands emphasized similarities to these countries concerning various attributes. These findings reinforce the idea that lesson-drawing is facilitated by a perception of similar national and policy contexts^4,5^. Ireland is an exception, as it was perceived to look at other English-speaking nations for lessons, both nearby and far away (The United Kingdom, Australia, and Canada), rather than close by countries with different languages. A previous country case-study indeed confirms that the Irish government engages in lesson-drawing and that they primarily look at other English-speaking nations^21^.

After dismissing lessons from global leader Australia (except for Ireland) because of perceived dissimilarities, countries seem to turn to ‘example countries’ within their own families. Our findings therefore roughly fall in line with the literature of ‘family of nations’, in which there is an English-speaking family (Ireland that looks to other English-speaking nations), a European Union family (Belgium and the Netherlands that predominantly look to other nearby European countries) and a Nordic family (Finland that looks to other Nordic countries)^11,13^. These families were hypothesized to look predominantly within their ‘families’ for lessons in tobacco control and therefore adopt similar policies, with policy convergence as a logical result^13^. Countries within the European Union are further accommodated to learn lessons within their family because of the EU as an institution, which serves as a platform to exchange (policy) ideas^13,23^. This function is perhaps comparable to that of the Nordic Council observed in our data. This council also seems to accommodate the exchange of (policy) ideas among the Nordic countries, further enabling lesson-drawing within the Nordic family. Our data suggest that the United Kingdom (and England in particular) serves as an example for tobacco control policy in Belgium, Ireland and The Netherlands. The UK thus serves as an example for other European countries, as it has relatively few remaining smokers and the most comprehensive set of policy measures enacted in Europe^2^, while at the same time importing policy instruments primarily from other, even more progressive English-speaking nations across the globe. In the study of Studlar^13^, the United Kingdom was indeed categorized as being part of both the English-speaking and the European Union group (so-called ‘overlapping’ families).

The finding that the German government is not perceived to draw lessons from different countries falls in line with a previous study of German tobacco control policymaking which concluded that German policymakers are largely self-sufficient in terms of health research and policymaking capacity, which results in more ‘inward-looking’ instead of ‘outward-looking’, which leads to less lessondrawing from other countries^24^. This disinclination may be related to a lack of political will^18^. Lessons are a means to a political end and their acceptance depends on the motive and opportunity of decision makers to translate them into domestic policy^25^. This suggests that lesson-drawing in itself may not be sufficient to explain policy change, in line with previous empirical findings^10^. Previous empirical work indeed suggests that, for example, the relative power of interest groups has an effect on tobacco control policy outcomes^22^. In Germany, tobacco control policymaking was more influenced by the tobacco industry than in Finland or Ireland^22^. In these latter countries, health organizations had more influence on the policy process^22^. Enforcement of Article 5.3 of the Framework Convention on Tobacco Control may be the solution in preventing such industry influence. In contrast to the German government however, the German NGO community was perceived to engage in lesson-drawing. NGOs frequently presented foreign examples, yet such examples did not find resonance with the government.

### Limitations

An important study limitation is that it is difficult to evaluate whether a lesson has actually been drawn or not. We presented and summarized considerations that policy participants perceive to play a role in national policy debates, but we have no data on whether a lesson is actually drawn or not, and there are no rigorous tools available to assess this^26^. Another study limitation is that we have interviewed a relatively small number of policy participants per country. However, as the selected interviewees play important roles in their national tobacco control policy environments (i.e. ‘elite interviewees’), we believe that together they voiced commonly held perceptions of national-level lesson-drawing processes.

Two other limitations concern the external validity of the findings. Firstly, policy participants only described marketing bans and smoke-free policies as policy instruments. It can be argued that different ‘example countries’ are mentioned when, for example, tax policies are considered. Different arguments to draw or dismiss lessons are then likely to arise as well. Border effects (such as cross-border cigarette purchasing) are observed when it comes to taxation policies^27^. A lesson from a country with similar border dynamics may then, for example, be more easily accepted. Secondly, all included countries were North-Western European. It is highly probable that in Eastern or Southern Europe, Asia, South or North America, other ‘example countries’ (or states) are selected, and other arguments are used to draw or dismiss lessons from them.

### Implications

Scientific evidence on the effectiveness of a policy measure is an important element of lessons to be drawn about tobacco policies^28^. A considerable body of research on the effectiveness and impact of tobacco control policies originates from countries within the English-speaking family of nations (the United States, Canada, Australia, and the United Kingdom), and to a much lesser extent from other parts of Europe^29^. As our findings suggest that governments may draw lessons more readily from other European nations nearby, it is important to invest more in European research on the effectiveness and implementation of tobacco control policies^30^.

While our article had focused on examples of lesson-drawing, we should not ignore the downside which is that most governments easily reject lessons from various other countries. Richard^4^ has identified a number of obstacles to lesson-drawing, including: a large scale of change needed to implement the policy instrument (e.g. introducing a new law as opposed to an amendment to an existing law), a complex cause and effect structure of the policy instrument (e.g. outcomes of the instrument that are unpredictable rather than predictable), and incongruities between policymakers’ values with those inherent to the instrument (e.g. policymakers that aspire a free market rather than a strong government). Scholars may assess whether such obstacles indeed play a role in rejecting foreign lessons in tobacco control.

Proponents of tobacco control may use our findings to facilitate successful lesson-drawing by choosing best practice examples in tobacco control from countries similar or close to their own country, or by emphasizing similarities in policy contexts to those of the global or European leaders in tobacco control.

## CONCLUSIONS

Countries in North-Western Europe seem to draw policy lessons based on proximity and similarity to other countries concerning marketing bans and smoke-free policies.

## Data Availability

The data supporting this research are available from the authors on reasonable request.
